# Evaluation of Hepatic Steatosis in Rodents by Time-Domain Nuclear Magnetic Resonance

**DOI:** 10.3390/diagnostics9040198

**Published:** 2019-11-20

**Authors:** João A.B. Pedroso, João Paulo Camporez, Luciana T. Belpiede, Rafaela S. Pinto, José Cipolla-Neto, Jose Donato

**Affiliations:** Departamento de Fisiologia e Biofísica, Instituto de Ciências Biomédicas, Universidade de São Paulo, São Paulo 05508-000, Braziljpcamporez@yahoo.com.br (J.P.C.); lucianabelpiede@hotmail.com (L.T.B.); nutrirafaelasilverio@gmail.com (R.S.P.); cipolla@icb.usp.br (J.C.-N.)

**Keywords:** obesity, hepatic steatosis, liver

## Abstract

Devices that analyze body composition of rodents by time-domain nuclear magnetic resonance (TD-NMR) are becoming popular in research centers that study metabolism. Theoretically, TD-NMR devices can also evaluate lipid content in isolated tissues. However, the accuracy of TD-NMR to determine hepatic steatosis in the liver of small laboratory animals has not been evaluated in detail. We observed that TD-NMR was able to detect increased lipid content in the liver of rats consuming high-fat diet (HFD) for 12 weeks and in genetically obese (*Lep^ob/ob^* and *Lepr^db/db^*) mice. The lipid content determined by TD-NMR showed a positive correlation with triglyceride content measured by colorimetric assays. In contrast, TD-NMR did not detect hepatic steatosis in C57BL/6 mice consuming HFD for 4 or 12 weeks, despite their obesity and increased liver triglyceride content. These findings indicate that tissue mass and the severity of hepatic steatosis affect the sensitivity of TD-NMR to detect liver lipid content.

## 1. Introduction

Hepatic steatosis, also known as fatty liver disease, is a condition of excessive lipid accumulation in the liver. There are several conditions that may promote hepatic steatosis, such as obesity, alcoholism, chemotherapy or infectious causes [1]. Non-alcoholic fatty liver disease (NAFLD) is a common spectrum of fatty hepatic steatosis [2], affecting 25% of adult individuals in the world, with elevated rates in South America (31%) and the Middle East (32%) [3]. NAFLD has a strong association with diverse hepatic health problems, such as cirrhosis or liver cancer [4], leading to reduction of the rate of survival in the long term [2]. The severe progressive form of NAFLD, non-alcoholic steatohepatitis (NASH), is becoming a top indication of liver transplant in the United States and represents a major cause of hepatocellular carcinoma [5]. Also, NALFD and NASH are major risk factors closely associated with the development of insulin resistance and type 2 diabetes [6,7,8]. Therefore, investigations about fatty liver disorders are essential for the development of potentially new treatment and/or prevention approaches.

Different methodological strategies are commonly used to study liver steatosis in animal models. The chronic consumption of a high-fat diet (HFD), excessive fructose intake or deficient ingestion of methionine and choline are example of dietary manipulations that can lead to fatty liver [9]. Some mutations that cause massive obesity are also useful to study the pathophysiological mechanisms of hepatic steatosis, including mice carrying loss of function of the leptin gene (*Lep^ob/ob^* mice) or the leptin receptor gene (*Lepr^db/db^* mice). These animals develop mild to severe steatosis by 12 weeks of age even in a regular chow diet [10].

In animal models, a fatty liver is evaluated by histological approaches, such as hematoxylin and eosin or Oil Red O staining, or by colorimetric assays. In both cases, the procedures require reagents and, sometimes, a considerable amount of time for the assessments. In addition, these methods evaluate small liver samples, which may not be representative of the overall hepatic fat content. Previous studies have shown that liver fat distribution is not homogeneous and the results from analyses using small samples may show considerable variability [11]. Therefore, the development of faster and cost-free methods that analyze the whole liver fat content is of interest.

In this sense, time-domain nuclear magnetic resonance (TD-NMR) instruments are becoming popular and they are available in many research centers around the world that study metabolic diseases in animal models. These instruments are used to analyze body composition enabling a non-invasive, fast and in vivo assessment of body fat and lean mass in small animals. The body composition results obtained using TD-NMR show good reproducibility in comparison with classical methods, such as chemical analysis of the carcass [12]. Notably, TD-NMR instruments can also measure the fat content of isolated tissues. However, to our knowledge there is poor information about the precision of TD-NMR to determine hepatic steatosis in comparison with gold-standard methods. Therefore, our objective was to investigate whether the measurement of hepatic fat content by TD-NMR equipment could represent an alternative procedure to study hepatic steatosis in rodent animals, especially when considering that this analysis could be performed very quickly and practically cost-free.

## 2. Material and Methods

### 2.1. Animals

Different rodent models were analyzed in the present study. In the first set of experiments, 3 weeks old male Wistar rats were allowed to eat either a regular rodent chow diet (3.85 kcal/g; 9.4% calories from fat; *n* = 20) or a high-fat diet (HFD; 5.41 kcal/g, 58.55% calories from fat; PragSoluções, Brazil; *n* = 20) for 12 weeks. In another experiment, 11-week-old *C57BL/6* male mice received either chow diet or HFD (5.24 kcal/g, 60% calories from fat; Research Diets, USA) for 4 or 12 weeks (*n* = 7 each group). The distinct durations in which animals received HFD were employed to evaluate mice with different degrees of obesity and hepatic steatosis [7,9]. Lastly, 16-week-old genetically obese mice (*Lep^ob/ob^*, *n* = 6 and *Lepr^db/db^*, *n* = 2) or wild-type mice (*n* = 10) receiving a regular rodent chow diet were studied. Animals were maintained under standard conditions of light (12 h light/dark cycle) and temperature (23 ± 2 °C). All animal procedures were approved by the Ethics Committee on the Use of Animals of the Institute of Biomedical Sciences, University of São Paulo (protocol numbers: 86/2016 and 73/2017; approval date: 7 July 2017).

### 2.2. Experimental Design

The rats were weighted and euthanized to determine body adiposity by measuring the masses of the perigonadal and retroperitoneal fat pads. In the groups of mice, body weight was determined weekly. Before euthanasia, total body fat was measured by TD-NMR using the LF50 body composition mice analyzer (Minispec; Bruker, Germany). TD-NMR equipment acquires radio frequency signals generated by the hydrogen spins from soft tissues such as adipose and muscle and uses the contrast in relaxation times of the hydrogen spins, or the amplitude, duration, and spatial distribution of these NMR signals from the different tissues to estimate body composition. Thus, animals were placed in a clear, plastic cylinder (50 mm diameter) and kept immobile by insertion of a tight-fitting plunger into the cylinder. The tube was then lowered into the sample chamber of the instrument for approximately 2 min, which is the duration of each scan. The TD-NMR equipment was calibrated according to manufacturer’s procedure. In all animals, the liver was dissected, weighted, wrapped in plastic and the fat content was measured by TD-NMR. A liver sample was collected and stored at −80 °C until total triglyceride (TAG) content was measured by colorimetric assay.

### 2.3. Hepatic Triglyceride (TAG) Content

To directly determine hepatic TAG content, hepatic lipids were extracted using the method of Folch et al. [13]. Briefly, this method involves two phases. The first step consists of extracting lipids and filtering the homogenate. In the second step, total lipid content is separated from non-lipid substances, collected and stored to further analyse. In this study, approximately 50 mg of liver tissue was weighed and homogenized in 1 mL of chloroform/methanol (2:1) solution. Then, the crude extracted was shaken at room temperature for 3–4 h to extract lipids into organic phase. Afterward, 200 µL of 1 M H_2_SO_4_ was added in the solution and centrifuged in low speed (2000 rpm) for 10 min to separate the aqueous (upper) and organic (lower) phase. The organic phase was carefully collected and dried overnight at room temperature. Lastly, the lipids extracted were reconstituted in 1 mL chloroform and stored at −20 °C. TAG content was measured by colorimetric assay using a commercially available kit (Triglycerides Liquiform–Labtest ref #87).

### 2.4. Statistical Analysis

All data were expressed as mean and standard error of the mean (s.e.m.). All statistical analyses and graph preparations were performed using GraphPad Prism 7.0 (GraphPad Software, San Diego, CA, USA). A two-tailed Student’s *t*-test was used to compare the differences between the groups. Pearson correlation coefficient was calculated to measure the correlation between the hepatic lipid content determined by TD-NMR and the TAG content determined by calorimetric assay. *p* values < 0.05 were considered statistically significant.

## 3. Results

### 3.1. Body Composition Analyzer Detects Hepatic Steatosis in Rats Treated with High-Fat Diet (HFD) for 12 Weeks

**Figure 1:**
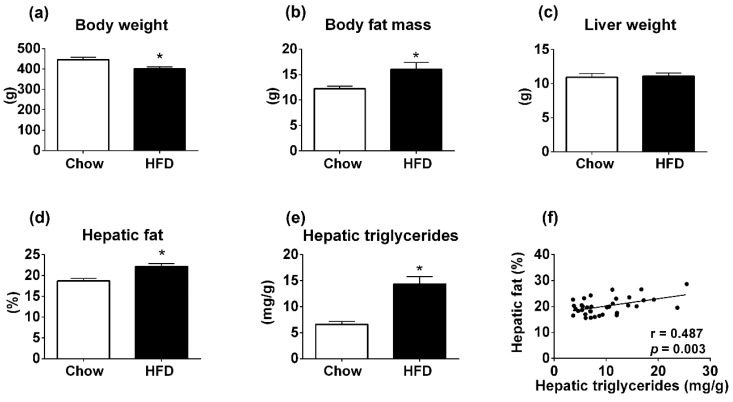
Metabolic parameters of Wistar rats consuming normal chow or high-fat diet (HFD) for 12 weeks. (**a**) Body weight (chow group, *n* = 20; HFD group, *n* = 15). (**b**) Body fat mass determined by summing the masses of the epididymal and retroperitoneal fat pads. (**c**) Liver weight. (**d**) Percentage of hepatic fat measured by time-domain nuclear magnetic resonance (TD-NMR). (**e**) Hepatic triglyceride (TAG) measured by calorimetric assay. (**f**) Linear correlation between hepatic fat measured by TD-NMR and hepatic TAG determined by colorimetric assay. * *p* < 0.05 versus chow group (Student’s *t*-test).

After 12 weeks, rats treated with HFD showed an unexpected reduction in body weight compared to control group receiving chow diet (*p* = 0.004; Figure 1a). However, the HFD group had increased body fat mass in comparison with the control group (*p* = 0.006; Figure 1b). These results demonstrate the effectiveness of HFD to increase body adiposity in rats. The liver weight showed no difference between the groups (*p* = 0.80; Figure 1c). Using TD-NMR, we observed a higher percentage of fat in the liver of the HFD group, compared to rats consuming a chow diet (*p* < 0.001; Figure 1d). The increased fat content in the liver of HFD group was confirmed by a well-established calorimetric assay that measures hepatic TAG content (*p* < 0.001; Figure 1e). In order to validate the use of TD-NMR to determine fat content in the liver, the percentage of fat in the liver measured by TD-NMR was correlated with hepatic TAG content (Figure 1f). Notably, we found a statistically significant and positive correlation between the percentage of fat in the liver measured by TD-NMR and TAG content determined by calorimetric assay (*r* = 0.48; *p* = 0.003; Figure 1f). These results demonstrate that the body composition analyzer that uses TD-NMR is sensitive to detecting hepatic steatosis in rats treated with HFD for 12 weeks.

### 3.2. Body Composition Analyzer Detects Hepatic Steatosis in Genetically Obese Mice Consuming Regular Rodent Chow

**Figure 2:**
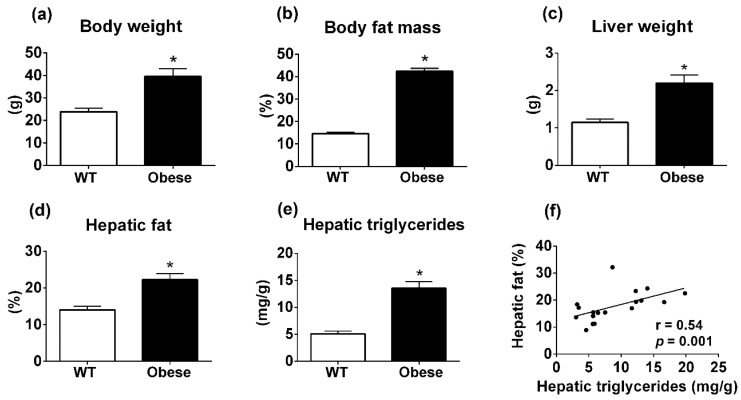
Metabolic parameters of wild-type or genetically obese *Lep^ob/ob^* and *Lepr^db/db^* mice. (**a**) Body weight (wild-type (WT), *n* = 10; Obese: *Lep^ob/ob^*, *n* = 6 and *Lepr^db/db^*, *n* = 2). (**b**) Body fat mass determined by TD-NMR. (**c**) Liver weight. (**d**) Percentage of hepatic fat measured by TD-NMR. (**e**) Hepatic TAG measured by calorimetric assay (**f**) Linear correlation between hepatic fat measured by TD-NMR and hepatic TAG determined by colorimetric assay. * *p* < 0.05 versus WT group (Student’s *t*-test).

To further investigate whether the body composition analyzer is sensitive enough to detect liver steatosis in smaller animals, we studied genetically obese mice that develop severe obesity due to the lack of leptin action [14]. *Lep^ob/ob^* and *Lepr^db/db^* mice were included in the same experimental group that was defined as the obese group and compared with age-matched C57BL/6 wild-type (WT) mice. As expected, obese group had higher body weight and percentage of body fat, compared to WT group (Figure 2a,b). Obese mice also had increased liver weight in comparison with WT group (*p* < 0.001; Figure 2c). In addition, the body composition analyzer detected increased percentage of fat in the liver of obese mice compared to WT animals (*p* < 0.001; Figure 2d). The higher hepatic steatosis in the genetically obese group was confirmed by measuring hepatic TAG content (*p* < 0.001; Figure 2e). Importantly, the percentage of fat in the liver determined by TD-NMR showed a positive and significant correlation (*r* = 0.54; *p* = 0001) with hepatic TAG content (Figure 2f). Therefore, the body composition analyzer can also detect hepatic steatosis in genetically obese mice consuming regular rodent chow.

### 3.3. Body Composition Analyzer is Not Accurate for Determining Hepatic Steatosis in Mice Fed HFD

**Figure 3:**
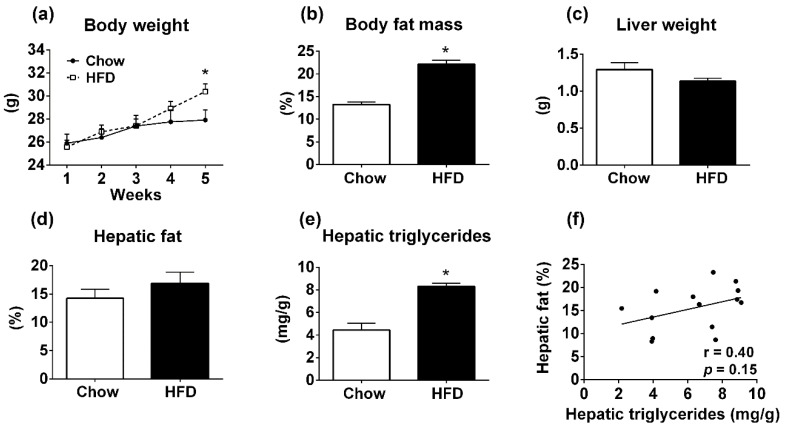
Metabolic parameters of C57BL/6 mice consuming normal chow or HFD for 4 weeks. (**a**) Body weight (chow group, *n* = 7; HFD group, *n* = 7). (**b**) Body fat mass determined by TD-NMR. (**c**) Liver weight. (**d**) Percentage of hepatic fat measured by TD-NMR. (**e**) Hepatic TAG measured by calorimetric assay. (**f**) Linear correlation between hepatic fat measured by TD-NMR and hepatic TAG determined by colorimetric assay. * *p* < 0.05 versus WT group (Student’s *t*-test).

Mice fed HFD for 4 weeks showed higher body weight compared to the control group receiving chow diet (*p* < 0.05; Figure 3a) and increased body fat mass (*p* < 0.001; Figure 3b). The liver weight showed no difference between the groups (*p* = 0.16; Figure 3c). Using TD-NMR, the HFD group showed a similar percentage of fat in the liver in comparison with the control group (*p* = 0.31; Figure 3d). However, increased hepatic TAG content was found in the HFD group compared to the control group (*p* < 0.001; Figure 3e). Consequently, there was no correlation between the percentage of fat in the liver measured by the body composition analyzer and the TAG content determined by the calorimetric assay (*p* = 0.15, *r* = 0.40; Figure 3f).

**Figure 4:**
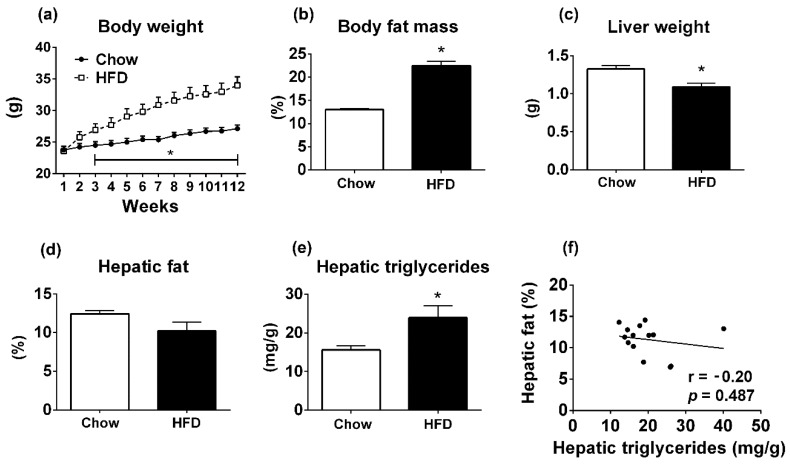
Metabolic parameters of C57BL/6 mice consuming normal chow or HFD for 12 weeks. (**a**) Body weight (chow group, *n* = 7; HFD group, *n* = 7). (**b**) Body fat mass determined by TD-NMR. (**c**) Liver weight. (**d**) Percentage of hepatic fat measured by TD-NMR. (**e**) Hepatic TAG measured by calorimetric assay. (**f**) Linear correlation between hepatic fat measured by TD-NMR and hepatic TAG determined by colorimetric assay. * *p* < 0.05 versus WT group (Student’s *t*-test).

We also investigated mice treated with HFD for 12 weeks. As expected, the HFD group showed higher body weight (*p* < 0.05; Figure 4a) and body fat mass (*p* < 0.001; Figure 4b), compared to the control group. However, the HFD group exhibited reduced liver weight compared to animals receiving chow (*p* = 0.002; Figure 4c). Despite these changes, no difference in the percentage of fat in the liver was observed between groups using TD-NMR (*p* = 0.09; Figure 4d). In contrast, TAG content determined by calorimetric assay was significantly higher in HFD mice compared to the control group (*p* = 0.002; Figure 4e). Consequently, there was no correlation between TD-NRM values and liver TAG content (*p* = 0.487; Figure 4e). Together, these data demonstrate that the body composition analyzer is not accurate for measuring hepatic steatosis in mice fed with HFD.

## 4. Discussion

Devices that analyze the body composition of rodents by TD-NMR are becoming increasingly popular and nowadays they are frequently available in many research centers around the world that study metabolic diseases in laboratory animals. The key advantage of equipment that employs TD-NMR, compared to other methods that also allow non-invasive, sequential longitudinal scans, such as dual-energy X-ray absorptiometry, micro-computed tomography or quantitative nuclear magnetic resonance [15], is its practicality and ease of use because each analysis takes less than 2 min and is performed in non-anaesthetized animals. Additionally, body composition results are readily available after the scan and do not require further analysis. Moreover, TD-NMR presents good reproducibility and accuracy in determining body composition compared to chemical analyses [12]. In the present study, we investigated whether commonly used rodent body composition analyzers that employ TD-NMR can detect hepatic steatosis as efficiently as well-established colorimetric assays. We observed that in some conditions, such as HFD in rats or in genetically obese mice, TD-NMR was able to detect accurately the higher fat content in the liver, exhibiting a significant and positive correlation with TAG content measured by calorimetric assays. However, hepatic steatosis was not detected by TD-NMR in C57BL/6 mice consuming HFD, despite their increased TAG content in liver.

HFD intake in rats and mice causes an increase in body fat favoring the development of several metabolic imbalances, particularly obesity [14]. The fat deposition not only increases in the white adipose tissue of animals fed HFD, but also ectopically, such as in liver and skeletal muscle [8,16,17]. The severity of hepatic steatosis depends on several factors, including the exposure time to HFD, the percentage of fat in the diet, the inclusion of other nutrients that may affect lipid metabolism as well as the genetic background and sex of the animal [9,18]. In both rats and mice, we observed that HFD intake increased body fat mass. However, the reduced body weight in the rats consuming HFD was an unexpected result. Previous studies have shown that HFD does not necessarily leads to weight gain in rats, even though it causes metabolic imbalances, such as glucose intolerance and fatty liver [19,20]. Thus, the increased adiposity of HFD rats may have been compensated by a decrease in lean mass, secondary to the dysfunctions caused by HFD intake. As expected, a higher TAG content in liver was observed in both rats and mice consuming HFD. In contrast, TD-NMR only detected hepatic steatosis in the rats. Although this divergent result could be caused by differences between species or diet composition (in the present study rats received a different HFD compared to mice), these variables cannot explain the insensitivity of the body composition analyzer to detect increased hepatic fat, especially because the mice clearly developed the expected obesity and the colorimetric assay detected increased TAG content in liver. Thus, we hypothesize that the size of liver may have played a role in the sensitivity to detect hepatic steatosis using TD-NMR. While the liver of rats had more than 11 g, the liver mass of mice was less than 1.5 g on average. Therefore, the sensitivity of this method increases as the sample size becomes bigger. In this regard, body composition analyzers seem to be useful in detecting hepatic steatosis in larger laboratory animals, such as rats. However, in mice it may be necessary to pool samples of several animals to be able to detect precisely the percentage of fat mass in liver.

Sample size cannot totally explain the lack of sensitivity to identify fatty liver in mice. When genetically obese mice were analyzed, TD-NMR was able to detect an increased percentage of fat in the liver showing a positive correlation with TAG content determined by colorimetric assay. *Lep^ob/ob^* and *Lepr^db/db^* mice are known to develop a marked hepatic steatosis, which is usually more severe than that found in mice consuming HFD for 4 or 12 weeks [9,21]. Therefore, it seems that when the hepatic steatosis is more severe, body composition analyzers are able to detect increased liver fat content, even in small samples.

Some methodological considerations are important. While TD-NMR seems to detect any sort of lipids in the samples [22], the colorimetric method used in the present work assesses TAG [23]. Although TAG is the most common form of lipids found in the organs [24], the differences in the compounds detected by these methods may decrease their correlation coefficient. In the present study, we used only one model of rodent body composition analyzer. However, different brands are commercially available and each manufacturer normally produces several models that allow the analysis of a wide variety of animals (based on the maximum weight). Thus, we cannot guarantee that other body composition analyzers will show the same sensitivity as the model used in the present study. Therefore, it will be important that validation experiments are performed in other equipment models before using TD-NMR to detect hepatic steatosis.

Metabolic diseases are a major health problem worldwide [25]. Hepatic steatosis is a common complication of obesity, although several other conditions can also lead to a fatty liver [1]. Since fatty liver is an important risk factor for the development of other complications, such as non-alcoholic steatohepatitis, diabetes, liver cancer and dyslipidemia [4], the identification of methods that allow a fast and cost-free measurement of hepatic liver content is of interest. In the present study, we showed that rodent body composition analyzers that employ TD-NMR can be used to detect changes in hepatic fat content. However, tissue mass and the severity of hepatic steatosis affect the sensitivity of TD-NMR to detect liver lipid content. Since our findings showed that hepatic steatosis could be detected in some situations, but not in others, we propose that this equipment should be used to provide a preliminary result in cases when the researchers are not initially prone to invest time and financial resources to analyze hepatic steatosis. Then, these preliminary data can be confirmed or validated with standard methods to assess fatty liver, such as colorimetric or histological assays. Thus, this type of equipment can increase the frequency by which liver steatosis is analyzed, contributing as an additional tool to improving the understanding of the pathophysiology of this condition, as well as how external or organic factors influence the development of fatty liver.
